# Role of CRH in colitis and colitis-associated cancer: a combinative result of central and peripheral effects?

**DOI:** 10.3389/fendo.2024.1363748

**Published:** 2024-03-28

**Authors:** Chao Zhu, Shengnan Li

**Affiliations:** Department of Pharmacology, School of Basic Medical Sciences, Nanjing Medical University, Nanjing, China

**Keywords:** CRH, CRF receptors, stress, colitis, colitis-associated colon cancer

## Abstract

Corticotropin-releasing factor family peptides (CRF peptides) comprise corticotropin releasing hormone (CRH), urocortin (UCN1), UCN2 and UCN3. CRH is first isolated in the brain and later with UCNs found in many peripheral cells/tissues including the colon. CRH and UCNs function via the two types of receptors, CRF_1_ and CRF_2_, with CRH mainly acting on CRF_1_, UCN1 on both CRF_1_ &CRF_2_ and UCN2-3 on CRF_2_. Compiling evidence shows that CRH participates in inflammation and cancers via both indirect central effects related to stress response and direct peripheral influence. CRH, as a stress-response mediator, plays a significant central role in promoting the development of colitis involving colon motility, immunity and gut flora, while a few anti-colitis results of central CRH are also reported. Moreover, CRH is found to directly influence the motility and immune/inflammatory cells in the colon. Likewise, CRH is believed to be greatly related to tumorigenesis of many kinds of cancers including colon cancer via the central action during chronic stress while the peripheral effects on colitis-associated-colon cancer (CAC) are also proved. We and others observe that CRH/CRF_1_ plays a significant peripheral role in the development of colitis and CAC in that CRF_1_ deficiency dramatically suppresses the colon inflammation and CAC. However, up to date, there still exist not many relevant experimental data on this topic, and there seems to be no absolute clearcut between the central and direct peripheral effects of CRH in colitis and colon cancer. Taken together, CRH, as a critical factor in stress and immunity, may participate in colitis and CAC as a centrally active molecule; meanwhile, CRH has direct peripheral effects regulating the development of colitis and CAC, both of which will be summarized in this review.

## 1 Introduction

Ulcerative colitis (UC) and Crohn’s disease, the common chronic inflammation in the gastrointestinal system, are the two main forms of inflammatory bowel disease (IBD) (1, 2). The precise cause of IBD is not thoroughly known yet. It is observed that UC patients may have a dysregulated mucosal immune response to commensal gut flora, resulting in bowel inflammation characteristically restricted to the mucosal surface in the colon (3, 4). Chronic inflammation is fundamentally an immune response, which provides microenvironment for tumorigenesis and accounts for a big portion of cancer-causing factors (4), which is in concert with the case between colitis and colorectal cancer (CRC) (5, 6), although meta-analysis does not show an increased CRC risk over time of inflammation (7). CRC is one of the most common forms of malignant tumor worldwide, and patients with UC are at higher risk for developing CRC, i.e., colitis-associated colon cancer (CAC), than the general population (8). Therefore, anti-inflammation treatment is likely a useful approach for preventing the occurrence of CAC (9). However, despite constant studies and advances in conventional and/or targeted therapy, the survival rate of CRC patients is still not very high (10, 11).

Corticotropin-releasing factor family peptides (CRF peptides) include 4 members, corticotropin releasing hormone (CRH), urocortin (UCN1), UCN2 and UCN3, mediating their effects via two distinct CRF receptor subtypes, CRF_1_ and CRF_2_, with CRH being the selective agonist of CRF_1_, UCN1 of both, and UCN2-3 of CRF_2_ (12–18) (**Table 1**).

**Table 1:** Corticotropin-releasing family peptides (CRF peptides) and receptors.

CRF peptides	Targeting Receptors
Corticotropin releasing hormone (CRH)	CRF_1_
Urocortin (UCN1)	CRF_1_, CRF_2_ (CRF_2α_, CRF_2ß_)
Urocortin 2 (UCN2)	CRF_2_ (CRF_2α_, CRF_2ß_)
Urocortin 3 (UCN3)	CRF_2_ (CRF_2α_, CRF_2ß_)

Both CRF_1_ and CRF_2_ belong to the seven transmembrane domain family positively coupled to adenylate cyclase via G proteins (13–15). CRH, a 41-amino acid peptide, is observed to activate cAMP/MAPK pathway via CRF_1_ (19, 20). It is recognized as a primary regulator of the hypothalamic pituitary axis (HPA axis) (17, 21–23). The paraventricular nucleus (PVN) of the hypothalamus is the main source for CRH in the brain (17). CRH, UCNs and the two receptors are also reported to express widely in peripheral cells/tissues, being recognized as important cardiovascular peptides and immune/inflammatory molecules (23–28). Their presence in gastrointestinal system has been detected for a long time since decades ago (29–31). Moreover, CRH and UCN3 are detected in the human colon (32) and UCN1 mRNA is observed in the rat enteric nervous system (33). CRF_1_ and CRF_2_ are encoded by specific genes (13). CRF_1_ is the main type of receptor in the brain (13) while it is also abundant in some peripheral cells/tissues including skin, inflammatory cells and gastrointestinal system (13, 28, 30, 34). And CRF_2α_ is predominantly found in neurons and CRF_2β_ in both brain and peripheral tissues including cardiac & skeletal muscle and the gastrointestinal tract (13, 35, 36). Both CRF_1_ and CRF_2_ are distributed within the rat colon: CRF_1_ is found in the colonic crypts, the surface epithelium, and the lamina propria of the proximal colonic mucosa. CRF_1_ expression is also detected in the myenteric and submucosal nervous plexus. CRF_2_ expression is found to be localized mainly in the luminal surface of the crypts and in blood vessels of the submucosal layer (31). Also in the human colonic mucosa, both CRF_1_ and CRF_2_ mRNA are detected in lamina propria mononuclear cells (30). These results support a role for the two receptors’ involvement in regulating peripheral colonic effects of CRH and UCNs. Since this review focuses on the selective CRF_1_ agonist, CRH, more about CRH/CRF_1_ effects will be discussed.

The hypothalamus-pituitary-adrenal (HPA) axis, functionally a hormone stimulating cascade, mainly CRH-adrenocorticotropin (ACTH)-cortisol axis, is a critical element for stress response and immune/inflammatory processes (37). Chronic stress, characterized by activation of HPA axis and sympathetic nervous system, has been reported to be an important reason in the development of inflammation and tumorigenesis (38–40), suggesting that CRH indirectly participate in inflammation and tumorigenesis via HPA axis as a centrally active molecule. Furthermore, HPA axis communicates with the immune system at multiple levels (41, 42). Bidirectional interactions between HPA and immunity contribute to their role in inflammation and cancers: HPA activation results in secretion of CRH, ACTH and cortisol modulating the immune response while immunity-related substances, such as interleukin-1 (IL-1), IL-6 and tumor necrosis factor alpha (TNF-α) can backwards stimulate the HPA axis (42). In addition, relationship between gut microbiome and the brain, i.e. brain-gut-microbiota axis, has attracted much attention for its complicated part in stress and IBD (43–45). The imbalance of brain-gut-microbiota axis also leads to dysregulation of the HPA axis (44). Therefore, it is reasonable that CRH, as the major mediator of stress response, may take a part in IBD and CRC via brain-gut-microbiota axis. Taken together, CRH is suggested to take part in colonic inflammation and inflammation-based tumorigenesis indirectly via HPA axis and brain-gut-microbiota axis.

Moreover, peripherally direct participation of CRH in colonic inflammation has been well proved (46, 47). The expression of CRH in the large bowel of patients with UC is found considerably enhanced in mucosal inflammatory cells and slightly increased in colonic mucosal epithelial cells, suggesting CRH’s role via modulating intestinal immune/inflammatory system in UC (47). Also, it is reported that CRH may induce intestinal hyperpermeability in human colon mucosa via mast cells (48). We and others have also reported the direct peripheral role of CRH/UCNs & receptors in immunity/inflammation and cancers (42, 49–54), including colitis and colitis-associated colon cancer (CAC) (55–58).

In summary, over recent decades, CRF peptides and receptors have been found to be significantly correlated with the bowel inflammation and the development of CAC. However, controversies over the origin of CRH action sites have always been existing. Up-to-date, taken together, it is understandable that CRH, as both a centrally active endocrine hormone and peripherally active peptide, may play an important role in colitis and CAC via both indirect actions regulating chronic stress and direct peripheral effects, although there still lack experimental evidences showing direct relationship between central CRH effect and colitis/CAC and only a few investigations show the direct peripheral effects of CRH on CAC.

## 2 The central role of CRH in colitis and CAC

Stress, inflammation and colon cancer are highly related, forming a CRH-system driven crosstalk (38). Therefore, there may not be an absolute clearcut between the central and peripheral effects of CRH on inflammation and cancer. Up to date, little evidence has suggested a direct relationship between central CRH and the development of colitis and CAC. Instead, the central role of CRH in inflammation and cancers is mainly thought to be via mediating HPA axis as a stress mediator (38, 59, 60). Under chronic stress, the HPA axis is activated and the release of CRH from the PVN of the hypothalamus at its nerve endings in eminence, which is carried to the pituitary gland through the portal vessel, stimulating the secretion of ACTH, which in turn stimulates the secretion of cortisol from the adrenal gland. The hypothalamus-released CRH acts on CRF_1_ in the pituitary gland, causing ACTH release from the anterior pituitary (13, 61). About half of CRH in the brain is found to be bound with CRH binding protein (CRH-BP). In exposure to stress, the expression of CRH-BP increases in a time-dependent fashion, likely being a negative feedback mechanism for CRH’s action on CRF_1_ (62).

### 2.1 Central role of CRH/CRF_1_ in colitis

There exist contrary reports about CRH’s role in colitis. As summarized in the following, some researchers observe no effect or anti-inflammatory effect while many others find its pro-inflammatory actions in colitis.

#### 2.1.1 The non-proinflammatory/anti-inflammatory role of central CRH in colitis

CRH is a 41-amino acid peptide, a primary regulator of the HPA axis and a coordinator of the gastrointestinal response to stress (22, 63, 64). The most important effect of central CRH/CRF_1_ is to stimulate the pituitary gland to release ACTH causing cortisol secretion of from the adrenal gland cortex, i.e., mediating the function of HPA axis (61). Since cortisol is an anti-inflammatory hormone, CRH is normally recognized to act in an anti-inflammatory fashion. However, only a few reports present consistent evidences in case of colitis. While acute colonic inflammation induced CRH secretion from PVN in the hypothalamus, CRH level is found to remain at a high level in the brain after the recovery of colitis (65), suggesting a weak link between central CRH effect and colitis. On the other hand, Gue et al. find that centrally injected CRH may have complicated influences on colitis (66). By evaluating the influence of stress and the involvement of CRH on experimental colitis in rats, it is observed that centrally injected CRH antagonist, alpha-helical CRH-(9-41) has no effect on trinitrobenzenesulfonic acid-induced colitis but enhances the effects of stress on colitis, suggesting that central CRH may only participate in controlling the process of colitis in case of stress (66). Moreover, Million et al. observe a protective role of brain CRH from stress-induced worsening of colitis (67). They assess the role of central CRH in stress-induced worsening of colitis in inbred rat strains with hypo (Lewis) and hyper (Fischer344) CRH responses to stress. It is observed that trinitrobenzenesulfonic acid induces colitis with similar severity in both strains, which is inhibited by central injection of CRH. Chronic stress aggravates colitis more in Lewis than Fischer rats, which is reversed by central injection of the CRH antagonist astressin, indicating that central CRH restrains the stress’ proinflammatory action in experimental colitis (67). Similarly, it is reported that central CRH inhibits gastric motility, which can also be abolished by the intracerebroventricular injection of astressin (68). However, its effect on the colon motility is the opposite, stimulating the movement and contributing to the process of colitis (68) (see below 1.1.2).

#### 2.1.2 The pro-inflammatory effect of central CRH in colitis as a stress mediator

Compiling evidences show that central CRH plays indirectly a proinflammatory role in colitis. HPA axis is the critical pathway of stress response, which is elicited by physical or psychological stimuli (stressors). A stress response involves activation of sympathetic-adreno-medullar (SAM) axis, HPA axis, and immune system, and a prolonged stressor exposure constitutes a chronic stress (69).

Chronic stress is known to promote IBD (64, 69), but the underlying mechanism remains largely unresolved. IBD is a model of microbial, immune and neuropsychological integration (70, 71). It is reported that chronic stress sensitizes mice to dextran sulfate sodium (DSS)-induced colitis and enhances the infiltration of proinflammatory cells in colonic lamina propria (72). Also, a marked increase in IL-6, a stress-inducible cytokine that further activates HPA axis in a positive feedback manner, is observed (73). Moreover, IBD is presumed to be a disorder of the brain–gut-microbiome link associated with exaggerated response to stress (74, 75). Under stress, inflammation-promoting bacteria expand while transferred gut microbiota from stressed mice facilitate DSS-induced colitis, which is abrogated by broad-spectrum antibiotic treatment (72). Therefore, it is obvious that chronic stress leads to colitis via disturbing gut microbiota and hence triggering immune system response. Based on these reports, CRH, as the main stress mediator, is evidently a critical factor in the development of colitis.

Interestingly, researchers record colonic motility and reveal that restraint stress, or intracerebroventricular injections of CRH, produce significant increases in colonic motility although CRH inhibits gastric motility (68, 76), which contributes to the occurrence of abdominal pain during IBD. Central injection of astressin is observed to block exogenous CRH action and colonic response to stress, showing an antagonistic action against CRH and stress-related alterations of gastrointestinal motor function, without an intrinsic effect in rats (68). Moreover, it is observed that the colonic contractions induced by central CRH are eliminated by intracerebroventricular pretreatment with astressin (76). On the other hand, peripherally administered CRH partially mimics the stress response of the gastrointestinal motility, exaggerated in IBD patients (77), further suggesting that CRH plays an important role in modulating brain-gut functions under stress.

CRH is also found to participate in IBD during acute stress. Zhao et al. establishes a model of psychosocial stress by peripheral administration of CRH and find that CRH aggravates DSS-induced colitis via the enhancement of intestinal macrophage autophagy (78). It is observed that peripherally used CRH aggravates the severity of DSS-induced IBD, increasing overall and local inflammatory reactions and infiltration. Under the IBD-related inflammatory challenges, the autophagy levels in intestinal macrophages are significantly increased, which is further enhanced by CRH. The autophagy inhibitor, chloroquine, markedly attenuates the detrimental effects of CRH reducing the severity and inflammatory reactions (78). These results may suggest that CRH, while working centrally mimicking stress, simultaneously exacerbates DSS-induced IBD via enhancing intestinal macrophage autophagy. Therefore, it is reasonable to believe that CRH and related receptors may be a potential therapeutic target for the treatment of IBD. Another investigation also shows that peripherally administered CRH mimic the effect of acute psychological stress, leading to increased intestinal permeability characterized in IBD (79). These findings further provide new insights into the complex interplay between the central and peripheral role of CRH in IBD since CRH is administered peripherally for stress model.

### 2.2 Central role of CRH in colitis-associated colon cancer

Rare evidence shows a direct relationship between the central CRH and the development of cancers. However, the relationship between chronic stress and tumor development has been frequently reported and widely reviewed (38–41, 80, 81). Therefore, it is believable that the central role of CRH in tumor is likewise mainly via the indirect way through HPA axis-mediated stress.

Clinically, chronic stress is found common among cancer patients due to stressors encountered (82). Primarily, chronic stress activates the classic neuroendocrine systems, the HPA axis and the SAM, whose continuous activations have been demonstrated to take part in cancer-promoting processes by altering the tumor microenvironment (TME) (39, 81, 83). Stress hormones can promote colon cancer development through a variety of mechanisms: 1) Corresponding changes in the body’s immune function and inflammatory response (40, 83, 84); 2) Significant influence on the gut flora, i.e. the brain-gut-microbiota axis, promoting the composition of pro-inflammatory microbiome and hence resulting in colitis leading to CAC (85, 86).

Although the mechanisms might be complicated, it is believable that the central CRH, the upper element of HPA axis and stress mediator, plays a role in CAC based on that central CRH participates in colitis (see 2.1) and the cross talks between inflammation/immunity and cancer (87, 88). Recently, the microbiota has been recognized as one of the key regulators of gut-brain function. Many factors, stress in particular, can influence microbiota composition (89). Importantly, dysbiosis of the gut microbiome is found to be associated with the development of colorectal cancer (90) (see below 2.2). As precedingly described, individuals having IBD develop more easily into CAC (7, 57), and gut microbiome is involved in colon inflammation and biosynthesis of chemical carcinogens such as N-nitroso compounds that drive carcinogenesis (90–92). Meta-analysis demonstrates that in patients under stress gut microbiota perturbations are associated with loss of certain anti-inflammatory bacteria but an enrichment of pro-inflammatory bacteria (89), suggesting an interaction between the central CRH and gut flora.

It is nowadays recognized that dietary mode is related to colon microbiota (92, 93), leading to the idea that modulating the growth of beneficial microbiota in the gut by dietary and life style interventions may be a useful approach for prevention of colon cancer (94, 95). Based on the importance of the brain-gut-microbe axis during chronic stress, interfering chronic stress using CRH-related drugs might also become a useful approach for CAC prevention and treatment.

## 3 Peripheral roles of CRH in colitis and CAC

As precedingly described, although CRH is first isolated in CNS where it is initially recognized to be the main target site, CRH and the other CRF peptides have then been observed existing and functioning peripherally. Furthermore, the two receptors, CRF_1_ and CRF_2_ have been detected in many types of peripheral cells/tissues, such as immune cells, endothelial cells, tumor cells, etc. (13, 28, 42). CRH and the other CRF peptides are revealed to have a variety of direct peripheral functions in cardiovascular system, gastrointestinal system and immune system (13, 42). Besides the centrally indirect influence via mediating stress, CRH is also reported by our group and others to play a direct peripheral role in inflammation and tumors, including colitis and CAC (13, 42, 43, 50, 57, 58).

### 3.1 Peripheral participation of CRH in colitis

CRH & UCNs and the receptors are observed to be closely related to gastrointestinal system (12, 13, 96, 97) and reported to be implicated in colitis (43, 98, 99).

Firstly, CRH, the selective CRF_1_ ligand/agonist, is reported to play a significant role in the gastrointestinal motility by stimulating enteric nervous system (100) and evidence supporting that peripheral CRH & CRF_1_ directly take part in brain-gut sensitization is increasing (43). As mentioned above, IBD displays chronic abdominal pain or discomfort due to altered gut motility and visceral sensation (1, 100). Moreover, peripheral injection of CRH or UCN1 inhibits human gastric emptying and motility through interaction with CRF_2_, but stimulates colonic motility through activation of CRF_1_ (101). CRH induces motility of the descending colon in both healthy subjects and colitis patients, the latter with greater motility indexes. Paralally, abdominal symptoms evoked by CRH in colitis patients last significantly longer than in healthy controls (101). Moreover, rectal electric stimulation-induced significantly higher motility indices of the colon in colitis patients (vs healthy controls) are suppressed after administration of the selective antagonist of CRH, alphahCRH (αhCRH). Consistently, αhCRH significantly reduces the ordinate scale of abdominal pain evoked by the electric stimulation in colitis patients without changing ACTH and serum cortisol levels (102). Interestingly, peripheral administration of CRH is observed to aggravate visceral sensorimotor function as well as ACTH response in IBD patients (43, 100, 103). However, αhCRH is found to suppress higher motility among IBD patients, reducing the abdominal pain without plasma ACTH & cortisol change (43), suggesting the dominant peripheral effect. Furthermore, αhCRH is observed to block colorectal distention-induced sensitization of the visceral perception in rats (43, 102, 104). All these results demonstrate that besides its central action, CRH/CRF_1_ enhances colon motility, contributing to abdominal pain peripherally.

Secondly, peripheral CRH may play a role in colitis via influencing immune/inflammatory processes (42). Our group find that expressions of UCN1 and CRH are enhanced in the colon of wild type (*Crhr_1_^+/+^*) mice during azoxymethane (AOM) and DSS treatment (57). CRF_1_ has a proinflammatory and therefore a carcinogenetic (see below) effect in the mouse colon. The extent and severity of inflammation are drastically decreased in *Crhr_1_^-/-^* mice with much lower inflammatory cytokines’ expression, grade of dysplasia and numbers of ulceration in the colon mucosa. Moreover, accompanying the markedly lower proinflammatory cytokines, IL-1, IL-6, and TNF-α, the anti-inflammatory factor, IL-10 is increased in *Crhr_1_^-/-^* mice. Our results are consistent with the reports that CRF_1_ activation promotes inflammation (13, 42, 50, 103). However, in case of innate immunity deficiency, the opposite effect of CRH/CRF_1_ is observed (98). Chaniotou group investigate the role of CRH in an innate immunity–dependent mouse model of IBD (98). *CRH^-/-^* mice are observed to have more colonic inflammation than *CRH^+/+^* mice in DDS-induced colitis model. Moreover, as precedingly described, it is observed that, CRH further enhances the promoted autophagy levels in intestinal macrophages in IBD patients, which is markedly attenuated by the autophagy inhibitor, chloroquine, reducing CRH-induced severity and inflammatory reactions (76). These results may suggest that CRH, while working centrally mimicking stress, simultaneously exacerbates DSS-induced IBD via enhancing intestinal macrophage autophagy. Therefore, it is reasonable to believe that CRH and related receptors may be a potential therapeutic target for the treatment of IBD. In addition, mast cells are found to be related to CRH effects participating in the process of colitis, increasing the intestinal mucosal permeability (101).

The role of CRF_2_ in colitis is also complicated. It is reported that CRF_2_ has a counter regulatory action against CRF_1_, maintaining a balance between CRF_1_ and CRF_2_ during inflammation (103). On one hand, CRF_2_ is observed to function as a proinflammatory element while on the other hand, it displays an anti-inflammatory feature (105). Activation of CRF_2_ is reported to promote inflammation during acute colitis but to inhibit inflammation during chronic colitis (106). In DSS-induced colitis, mucosal repair is delayed after administration of a CRF_2_ antagonist (106). Moreover, CRF_2_ is down-regulated in human colitis (107). Taken together, a balance between CRF_1_ and CRF_2_ may decide the process of inflammation (103). This balance-theory may well interpret that both CRF_1_ and CRF_2_ are found to participate in acute inflammation while CRF_2_ is the main type for repair (103). The theory may lead to better understanding the pathophysiology and provide novel therapeutic options targeting altered signaling balance of CRF_1_ and CRF_2_ in IBD.

### 3.2 Peripheral CRH’s role in colitis-associated colon cancer

CRH is present in the colonic mucosa of UC patients and acts as a proinflammatory factor modulating the intestinal immune system (29, 47). Furthermore, UCN1, the unselective agonist for CRF_1_ and CRF_2_, is found to be synthesized and secreted in plasma cells, related to the inflammation in colonic mucosa (108). In addition, in DSS-induced mouse colitis, CRF_1_ deficiency is observed to contribute to the relief of colon inflammation (57). These reports suggest that direct activation of CRF_1_ exerts an effect of exacerbating colitis and hence may promote CAC. Up-to-date, only a few experimental reports have been presented on the direct peripheral role of CRH & UCNs in CAC.

In 2014, we first investigate the functions of CRF_1_ signaling on the development of CAC by using CRF_1_ deficient mice in AOM and DSS-induced CAC model. And the results show that in WT (*Crhr_1_^+/+^*) mice, CRF_1_ and its endogenous ligands (UCN1 and CRH) are significantly enhanced in the colon during AOM and DSS treatment. Interestingly, in *Crhr_1_^-/-^* mice, tumorigenesis is dramatically reduced, accompanied by lower inflammatory responses, i.e., decreased IL-1β, IL-6, TNF-α level and macrophage infiltration. Moreover, a reduced activation of NF-κB and STAT3 phosphorylation, together with decreased proliferating & enhanced apoptotic cells in the colon are observed (57). The pro-tumorigenesis effect is further confirmed by our *in vitro* experiments (58). CRH enhances colon cancer cell proliferation, promoting colony formation. Furthermore, tube formation assay shows that CRH treatment significantly promotes angiogenesis of HUVECs. Further investigation shows that CRH/CRF_1_ significantly upregulates IL-6 and VEGF level through activating NF-κB. And the VEGF silence abolishes the tube formation induced by CRH. The CRH-induced IL-6 promotes STAT3 phosphorylation, whose inhibition by Stattic significantly inhibits the CRH-induced cell proliferation (58). Our data is consistent with a newly reported experiment, demonstrating that CRF_1_ deficiency inhibits CRC in AOM/DSS model (56). Taken together, CRH/CRF_1_ signaling promotes human colon cancer cell proliferation via NF-κB/IL-6/STAT3 and tumor angiogenesis via NF-κB/VEGF signaling pathway. Our results provide evidence to support a critical role for the CRH/CRF_1_ signaling in colon cancer progression and suggest its potential utility as a new therapeutic target for CAC. Based on the above, it is believable that CRH/CRF_1_ has a proinflammatory and therefore a pro-tumorigenic effect in terms of CAC, which might be a direction for developing new therapeutic approaches for inflammation and CAC prevention & treatment.

It is observed by Baritaki group that human colon tissues from CRC patients and CRC cell lines show decreased CRF_2_ expression (109). Contrary to CRH/CRF_1_, UCN2/CRF_2_ signaling inhibits cell proliferation, migration, invasion and colony formation. Furthermore, IL-1β, IL-6 and IL-6R mRNAs are diminished in CRC-CRF_2_+ cells. In CRC patients’ colon samples, CRF_2_ mRNA expression is inversely correlated with IL-6R (109). These results are in concert with the report that CRF_2_ deficiency worsens CRC in AOM/DSS model (56). However, opposite effect of CRF_2_ is also reported, i.e. CRF_2_ may promote the development of CRC (110). Also, a blood sample analysis suggests that CRF_2_ represent a risk factor for CRC development in Mexican patients (111), which raises a controversial question as well. Recently, researchers have reported the methylation status of both CRF_1_ and CRF_2_, and point out that this examination may become a promising screening approach for CAC (112, 113).

In addition, it is well established that there exists a link between gut microbiota and colitis & colon cancer (87, 114–116). As precedingly described, CRH exerts an effect on gut flora mainly as a central stress mediator via brain-gut axis. However, up-to-date, rare experimental evidence shows direct peripheral effects of CRH on gut flora.

## 4 Summary

Emerging evidence suggests that uncontrolled inflammation is a major risk factor for the development of cancer. A typical example for inflammation and inflammation-based tumor is colitis and CAC, strongly supported by the fact that patients with UC have a much higher risk for CAC. This review aims to mainly summarize the reports about CRH’ roles in the development of colitis and CAC, both central and peripheral, hoping to be helpful in giving a clue to future drug design of CRH relevance, as having been studied (55).

**Figure 1:**
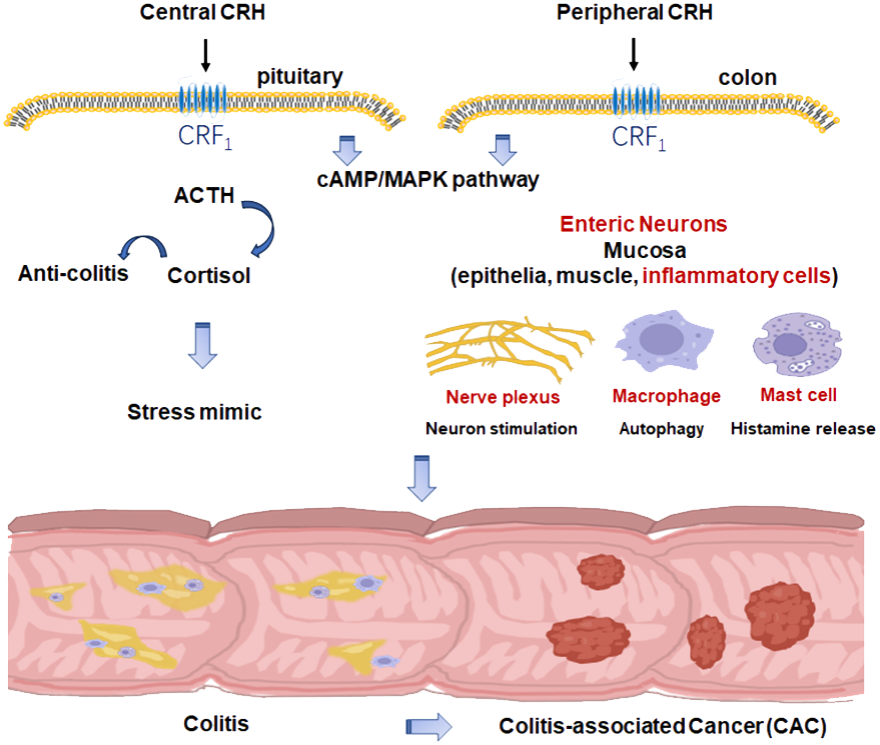
The role of CRH in colitis and CAC.

As summarized in **Figure 1**, CRH, as the main stress mediator, may participate in colitis and CAC via CRF_1_ as a central factor. Meanwhile, CRH and UCNs have been proved to play an important role in the development of colitis and CAC peripherally in which CRF_1_ may dominantly function as a pro-inflammatory and pro-tumorigenesis element while CRF_2_ may do oppositely. However, there exists no clearcut between CRH’s central and peripheral effects in colitis and CAC because of cross-talks between HPA axis and the immune system, and also between central and myenteric neurons.

However, there still lack experimental evidences for a direct relationship between central CRH and colitis/CAC, while there are also relatively few investigations on CRH’s peripheral effects on CAC. Given that CRH has a crucial role in stress and gastrointestinal system, with further evidence-reveal in the future, CRH may become a promising therapeutic target for colitis and CAC.

## Author contributions

CZ: Writing – original draft, Writing – review & editing. SL: Writing – original draft, Writing – review & editing.
